# Novel compound heterozygote mutations of *TJP2* in a Chinese child with progressive cholestatic liver disease

**DOI:** 10.1186/s12881-019-0753-7

**Published:** 2019-01-18

**Authors:** Ting Ge, Xinyue Zhang, Yongmei Xiao, Yizhong Wang, Ting Zhang

**Affiliations:** Department of Gastroenterology, Hepatology, and Nutrition, Shanghai Children’s Hospital, Shanghai Jiao Tong University, Shanghai, 200062 China

**Keywords:** Autosomal recessive disorder, Child, Compound heterozygote mutations, Progressive cholestatic liver disease, *TJP2*

## Abstract

**Background:**

Progressive familial intrahepatic cholestasis (PFIC) is a group of genetic autosomal recessive disorders that predominantly affects young children and results in early-onset progressive liver damage. Several types of PFIC were defined based on different genetic aetiologies in last decades.

**Case presentation:**

Here, we report a Chinese young child diagnosed as PFIC with variants in tight junction protein 2 (*TJP2*). The patient was affected by a long history of jaundice, pruritus, and failure to thrive. Highly elevated level of serum total bile acid (TBA) and normal levels of gamma-glutamyltransferase (GGT) were observed at hospitalization. The patient’s clinical symptoms could be alleviated by administration of ursodeoxycholic acid. Genetic testing by next generation sequencing (NGS) found novel compound heterozygote mutations c.2448 + 1G > C/c.2639delC (p.T880Sfs*12) in *TJP2*, which were inherited from her mother and father, respectively. Both mutations were predicted to abolish TJP2 protein translation, and neither has previously been identified.

**Conclusion:**

We report a Chinese female PFIC child with novel compound heterozygous mutations of *TJP2*. Genetic testing by NGS is valuable in the clinical diagnosis of hereditary liver disease.

**Electronic supplementary material:**

The online version of this article (10.1186/s12881-019-0753-7) contains supplementary material, which is available to authorized users.

## Background

Progressive familial intrahepatic cholestasis (PFIC), first described in 1969, is a group of genetic autosomal recessive disorders that are characterized by fluctuating jaundice, persistent cholestasis, pruritus and malabsorption [1, 2]. PFIC predominantly affects young children and results in early-onset progressive liver damage [2]. Persistent or repetitive cholestasis leads rapidly to end stage liver disease in untreated individuals with PFIC [2]. Several different genetic aetiologies have been linked to PFIC in last decades. PFIC 1 is caused by mutations in ATPase class I type 8B member 1 (*ATP8B1*) gene, which encodes the aminophospholipid flippase familial intrahepatic cholestasis-1 protein (FIC1) [3]. The mutant gene responsible for PFIC 2 is the ATP binding cassette family B member 11 (*ABCB11*) encoding the bile salt excretion protein (BSEP) [4]. Mutations in adenosine triphosphate-binding cassette subfamily B member 4 (*ABCB4*), which encodes the multidrug resistance P-glycoprotein 3 protein (*MDR3*) leads to the development of PFIC 3 [5]. Genetic mutations in tight junction protein 2 (*TJP2*) are linked to PFIC 4 with low or normal levels gamma-glutamyltransferase (GGT), and most of reported cases were homozygous mutations [6]. In addition, mutations in nuclear receptor subfamily 1, group H, member 4 (*NR1H4*), which encodes the farnesoid X receptor (FXR), were recently identified as responsible for a new type of PFIC [7]. However, known genes mutations still cannot account for all PFIC cases. Genetic profiling of children with cryptogenic cholestasis should be considered in clinical practice. Here, we report a young PFIC child with novel compound heterozygous mutations of *TJP2* in China. The clinical features and genetic variants of the patient are described in the study.

## Case presentation

A 23-month-old girl was admitted to our hospital because of a history of jaundice for more than one year. The patient was born at 30 weeks of gestation, with particular facial features including double pointed ears, slightly protruding jaw and hollow-eyed. Elevated levels of total serum bilirubin (TB), direct bilirubin (DB), and total bile acid (TBA) of the subject were observed since the age of 6 months (Table 1). The patient had low weight of 9.8 kg (P12) and height of 76 cm (P0) when hospitalized. Mild jaundice of the skin, moderate jaundice of the sclera, and pruritus were observed. Physical examination found a liver palpable 4.5 cm below the right costal margin. Spleen was 2 cm below the left costal margin. No signs were found that the lungs and heart are affected. Her hepatobiliary radionuclide imaging showed biliary obstruction and ultrasound indicated hepatomegaly, and magnetic resonance cholangiopancreatography (MRCP) was normal. The chest x-ray showed a normal thoracic spine, and there was no abnormality in cardiac ultrasound. The liver biochemical profile at age of 23 months revealed elevated ALT 147 U/L (5–40), AST 112 U/L (8–40), TB 70.08 μmol/L (3.40–17.10), DB 35.00 μmol/L (0–6.8), and TBA 203 μmol/L (0–10) (Table 1). Slightly low levels of vitamins were detected (Table 1). Laboratory tests showed a normal blood test, normal immunoglobulin G (IgG), IgA, IgM, and IgE levels. Lymphocyte subsets analysis was normal. Blood coagulation function, trace elements, Alpha fetal protein (AFP), blood tandem mass spectrometry and urine reducing substances were normal. Alpha-1-antitrypsin phenotype, serum amino acids, pathogens of Epstein Barr virus (EBV), TORCH, hepatitis A, B, C, E were all negative (data not shown). The patient was diagnosed as PFIC by manifestations of fluctuating jaundice, persistent cholestasis, pruritus and growth retardation. After admission, the patient was treated with oral ursodeoxycholic acid (25 mg/kg per day), compound glycyrrhizin tablet (2.5 mg/kg per day) and fat-soluble vitamins (vitamin k, 1 mg/kg, 3 times per day; vitamins AD, 1500 U per day; vitamin E, 10 mg/kg per day) for 2 weeks. The jaundice and pruritus were alleviated and liver function indexes were reduced, however, the level of TBA was still highly elevated (Table 1).

**Table 1:** Liver function index and routine clinical chemistry

Biochemical indices	Reference	6-month	12-month	23-month	23.5-month
TB (μmol/L)	3.40~17.10	145	27.83	70.08	37.16
DB (μmol/L)	0~6.8	85.5	16.28	35	17.30
ALT (U/L)	5~40	41	93	147	67
AST (U/L)	8~40	57	90	112	76
TBA (μmol/L)	0~10	200	227	203	299
GGT (U/L)	7~32	32	21.9	39	32
TG (mmol/L)	0–1.7			4.71	2.73
Cholesterol (mmol/L)	0–5.72			8.68	4.61
Vitamin A (μmol/L)	0.52~2.20			0.53	
Vitamin D (μmol/L)	25.00~200.00			25.39	
Vitamin E (μg/ml)	10.00~15.00			8.43	

*TB* total serum bilirubin, *DB* direct bilirubin, *ALT* alanine aminotransferase, *AST* aspartate aminotransferase, *GGT* gamma-glutamyltransferase, *TBA* total serum bile acid, *TG* triglyceride

## Genetic testing results

A total of 396 genes (Additional file 1: Table S1) associated with hereditary liver diseases based on OMIM (Online Mendelian Inheritance in Man, http://omim.org) were selected for development of a panel to screen genetic variations of the subject by next generation sequencing (NGS). Genomic DNA extracted from peripheral blood was fragmented by Q800R Sonicator (Qsonica) to generate 300–500 bp insert fragments. Custom designed NimbleGen SeqCap probes (Roche NimbleGen, Madison, Wis) were used for in-solution hybridization to enrich target sequences. Enriched DNA samples were indexed and sequenced on a NextSeq500 sequencer (Illumina, San Diego, Calif) with 100–150 cycles of single end reads. Primary data came in fastq form after image analysis and base calling was conducted using the Illumina Pipeline. Sequencing reads were mapped to the reference human genome version hg19 (2009–02 release, http://genome.ucsc.edu/) after removing adapters and low quality reads. Nucleotide changes observed of aligned reads were called and reviewed by using NextGENe software (SoftGenetics, State College, Pa). Large exonic deletions and duplications were screened by coverage-based algorithm developed in-house and eCNVscan. To detect copy number variants (CNVs), the normalized coverage depth of each exon of a test sample is compared with the mean coverage of the same exon in the reference file. Sequence variants were annotated using population and literature databases including 1000 Genomes, dbSNP, GnomAD, Clinvar, HGMD and OMIM. PolyPhen-2 [8] and SIFT [9] were used to analyze the structure of the protein, predict the conservation domain, function domain and perform the multiple sequence alignment. Variants interpretation was manipulated according to the American College of Medical Genetics and Genomics guidelines (ACMGG) [10]. The genetic tests and bioinformatics analysis were carried out by AmCare Genomics Laboratory, China.

**Fig. 1:**
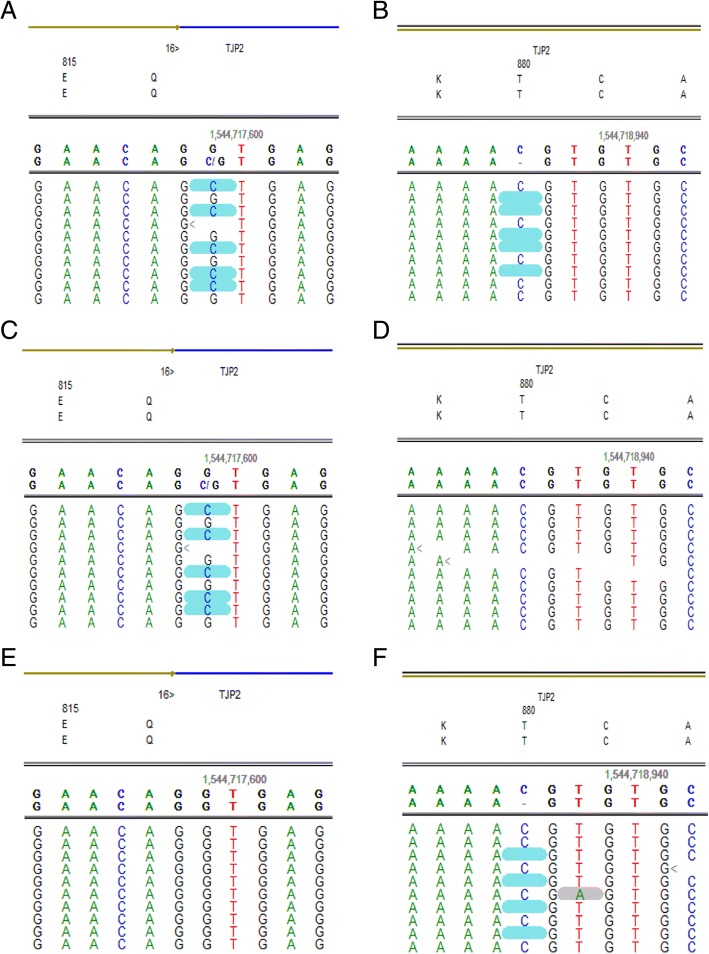
*TJP2* mutations analysis of the family. The proband carried compound heterozygous mutations c.2448 + 1G > C/c.2639delC (p.T880Sfs*12) of *TJP2* (**a**, **b**), c.2448 + 1G > C inherited from her mother (**c**, **d**), and c.2639delC (p.T880Sfs*12) from her father (**e**, **f**)

**Fig. 2:**
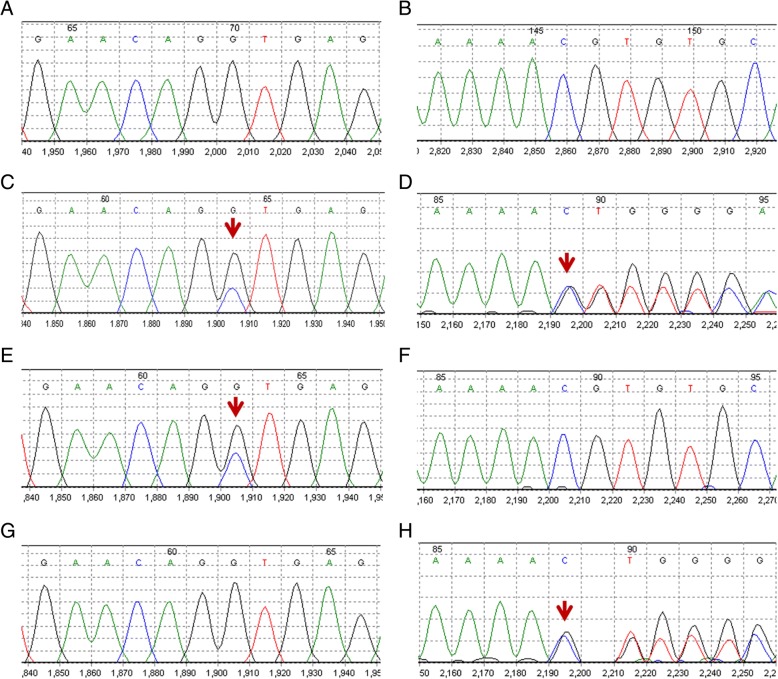
*TJP2* mutations confirmed by Sanger sequencing. **a**, **b** Healthy control without mutation. **c**, **d** The proband carried compound heterozygous mutations c.2448 + 1G > C/c.2639delC (p.T880Sfs*12) of *TJP2*. **e**, **f** The mother carried c.2448 + 1G > C mutation of *TJP-2* (**e**). **g**, **h** The father carried c.2639delC (p.T880Sfs*12) mutation of *TJP-2* (**h**)

Targeted genetic analysis on the proband identified two heterozygous variants in *TJP2*: a nucleotide substitution in splice site of exon 16, c.2448 + 1G > C, and a nucleotide C deletion in exon 17, c.2639delC (p.T880Sfs*12) (Fig. 1a, b). *In silico* predictors predict both mutations to be detrimental to *TJP2* transcripts and protein translation. Mutation c.2448 + 1G > C is located in highly conserved splice site and predicted to affect mRNA splicing of *TJP2* transcripts. The variant c.2639delC (p.T880Sfs*12) results in a frame-shift at amino acid 880 and is predicted to truncate *TJP2* transcription and translation by introducing a termination codon in exon 17. Both mutations were defined as damaging/likely pathogenic according to ACMGG [10], and have not been described previously. Gene sequencing on the unaffected parents showed the splicing mutation c.2448 + 1G > C in the mother (Fig. 1c, d), and the c.2639delC (p.T880Sfs*12) mutation in the father (Fig. 1e, f). No mutations in *ATP8B1*, *ABCB11*, *ABCB4*, and *NR1H4*, which linked to PFIC, were found. In addition, *TJP2* variants detected by NGS were confirmed by Sanger sequencing (Fig. 2).

## Discussion and conclusions

PFIC is a rare genetic autosomal recessive hereditary disorder with an incidence of 1: 50000 to 1: 100000 births [11]. Mutations in several genes encoding proteins involved in bile acid transportation have been demonstrated as causes of PFIC [2]. In this report, we described a female Chinese PFIC case with classical presentation of persistent jaundice, cholestasis, pruritus and severe growth retardation in the neonatal period. Elevated levels of serum bile acid and slightly low levels of GGT were presented in the patient. The patient showed severe growth retardation with low weight and height when admitted in our hospital at the age of 23 months. Gene panel test including 396 genes related with hereditary liver diseases identified novel compound heterozygous mutations c.2448 + 1G > C/c.2639delC (p.T880Sfs*12) of *TJP2* in the patient, which were inherited from her mother and father, respectively. *TJP2* variants detected by NGS were confirmed by Sanger sequencing.

*TJP2* is located on the long arm of chromosome 9, which encodes TJP2 protein, also known as zona-occludens 2 (ZO-2) [12]. TJP2 is a cytoplasmic component of cell-cell junctional complexes expressed in most of epithelial cells [12]. In 2003, mutation in *TJP2* associated with liver disease was reported in patients of Amish descent with familial hypercholanaemia (FHC) [13]. FHC is atypical for a liver disease that usually manifests only with pruritus and elevated serum bile acids, and the test results of biochemical markers of liver injury are normal [13]. Extrahepatic manifestations related with *TJP2* deficiency have been identified. It has been shown that heterozygous duplication and overexpression of TJP2 led to altered expression of apoptosis genes in a pedigree with progressive non-syndromic deafness [14]. The relevance of *TJP2* to PFIC was highlighted in 2014, when several homozygous missense mutations were identified in 12 children from 8 families affected with severe cholestatic liver disease [6]. It was shown that the protein-truncating mutations in the *TJP2* caused failure of protein localization and disruption of tight-junction structure, which led to a leakage of the biliary components through the paracellular space into the liver parenchyma [6]. Among those 12 children, 9 have required liver transplantation, 2 had stable liver disease with mild portal hypertension, 1 had recurrent unexplained hematomas, and 1 had poorly characterized lung disease [6]. Three reported pediatric HCC cases with *TJP2* mutations (1homozygosity of c.817delG, 1 compound heterozygous mutations of c.2668-1G > T/c.2438dupT, and 1 homozygous deletion of exon 18) were affected by very early onset of jaundice with normal range of GGT, 2 have required liver transplantation, and 1 was dead [15, 16]. Differently with pediatric TJP2 deficiency cases, several late onset adult patients with *TJP2* heterozygous mutations presented high level of GGT [17]. Furthermore, heterozygous mutations of *TJP2* were also reported in intrahepatic cholestasis of pregnancy (ICP) adult patients [18]. Those studies highlight the variability of clinical presentation in patients having mutations in *TJP2*. Here, we reported a case of PFIC with novel compound heterozygous mutations of *TJP2*. Slightly low levels of GGT were found in the patient, which were consistent with the previous reported *TJP 2* mutations causing PFIC 4 [6]. Both mutations are predicted to abolish TJP2 protein translation, and neither has previously been identified. Animal studies have shown that lacking of ZO-2 protein led to early embryonic lethality of mice, suggesting differences in redundancy in junctional complexes between organs and species [19]. The patient was treated with oral ursodeoxycholic acid, compound glycyrrhizin tablet and fat-soluble vitamins. The jaundice and itching were alleviated and liver function indexes were reduced, however, the level of TBA was still highly elevated. As *TJP2* mutations may be a risk factor for the development of HCC, the subject should be monitored closely in follow-up.

In summary, we for the first time report a Chinese female PFIC child with novel compound heterozygous mutations of *TJP2*. Genetic testing by NGS is valuable in clinical diagnosis of hereditary liver disease.

## Additional file

Additional file 1: **Table S1.** A list of screened genes. (XLSX 18 kb)

## Abbreviations

*ABCB11*: ATP binding cassette family B member 11
*ABCB4*: Adenosine triphosphate-binding cassette subfamily B member 4
AFP: Alpha fetal protein
ALT: Alanine aminotransferase
AST: Aspartate aminotransferase
*ATP8B1*: ATPase class I type 8B member 1
BSEP: Bile salt excretion protein
DB: Direct bilirubin
EBV: Epstein Barr virus
FHC: Familial hypercholanaemia
FIC1: Intrahepatic cholestasis-1 protein
FXR: Farnesoid X receptor
GGT: Gamma-glutamyltransferase
HCC: Hepatocellular carcinoma
ICP: Intrahepatic cholestasis of pregnancy
Ig: Immunoglobulin
MRCP: Magnetic resonance cholangiopancreatography
NGS: Next generation sequencing
*NR1H4*: Nuclear receptor subfamily 1, group H, member 4
PFIC: Progressive familial intrahepatic cholestasis
TB: Total serum bilirubin
TBA: Total bile acid
*TJP2*: Tight junction protein 2
ZO-2: Zona-occludens 2
